# Mechanical and leakage integrity testing considerations for evaluating the performance of tissue containment systems

**DOI:** 10.1038/s41598-023-31847-7

**Published:** 2023-03-29

**Authors:** Alexander Herman, Nandini Duraiswamy, Poulomi Nandy, Veronica Price, George Gibeily, Prasanna Hariharan

**Affiliations:** Center for Devices and Radiological Health, Food & Drug Administration, Silver Spring, MD USA

**Keywords:** Biomedical engineering, Mechanical engineering, Cancer prevention

## Abstract

Tissue containment systems (TCS) are medical devices that may be used during morcellation procedures during minimally invasive laparoscopic surgery. TCS are not new devices but their use as a potential mitigation for the spread of occult malignancy during laparoscopic power morcellation of fibroids and/or the uterus has been the subject of interest following reports of upstaging of previously undetected sarcoma in women who underwent a laparoscopic hysterectomy. Development of standardized test methods and acceptance criteria to evaluate the safety and performance of these devices will speed development, allowing for more devices to benefit patients. As a part of this study, a series of preclinical experimental bench test methods were developed to evaluate the mechanical and leakage performance of TCS that may be used in power morcellation procedures. Experimental tests were developed to evaluate mechanical integrity, e.g., tensile, burst, puncture, and penetration strengths for the TCS, and leakage integrity, e.g., dye and microbiological leakage (both acting as surrogates for blood and cancer cells) through the TCS. In addition, to evaluate both mechanical integrity and leakage integrity as a combined methodology, partial puncture and dye leakage was conducted on the TCS to evaluate the potential for leakage due to partial damage caused by surgical tools. Samples from 7 different TCSs were subjected to preclinical bench testing to evaluate leakage and mechanical performance. The performance of the TCSs varied significantly between different brands. The leakage pressure of the TCS varied between 26 and > 1293 mmHg for the 7 TCS brands. Similarly, the tensile force to failure, burst pressure, and puncture force varied between 14 and 80 MPa, 2 and 78 psi, and 2.5 N and 47 N, respectively. The mechanical failure and leakage performance of the TCS were different for homogeneous and composite TCSs. The test methods reported in this study may facilitate the development and regulatory review of these devices, may help compare TCS performance between devices, and increase provider and patient accessibility to improved tissue containment technologies.

## Introduction

Laparoscopic power morcellators are medical devices used during minimally invasive hysterectomy and myomectomy procedures to facilitate the removal of uterine fibroids and/or uterine tissue and continue to carry the advantages of lower complication rates and quicker recovery times as compared to open procedures^1–3^. Food and Drug Administration (FDA) issued a series of safety communications since 2014, highlighting the risk of unidentified uterine malignancy spreading to other organs during an uncontained power morcellation procedure^4,5^. Subsequently, to minimize the risk of spreading the cancerous cells during power morcellation, some studies have suggested the use of a tissue containment system (TCS) in patients^6–11^, which surrounds the power morcellator and the tissue to be excised and forms a barrier between them and the abdomen. As of April, 2022, the FDA has authorized marketing of two TCSs that are indicated for power morcellation for select patient populations through the De Novo classification process^12–14^ and one TCS via 510(k) process^15^.

The TCSs typically made from polymer-based materials help to contain and prevent penetration of tissue cells through the containment system wall. Several clinical studies have evaluated the integrity and applicability of these containment systems during clinical procedures^7,10,11,16–32^. Some of these studies have reported evidence for damage and/or leakage (or spillage) during surgery^16,20,21,24,26,27,29,30^. The potential causes for containment system failures include, but are not limited to, permeable containment system material, puncture by surgical instrument, tear due to excessive pressure, etc. Mechanical and leakage integrity of the TCS material is critical to ensure that the TCS maintains its barrier properties throughout the surgical procedure.

There are some open-access information available for the FDA-authorized^15,33,34^ devices that outlines the type of mechanical and leakage integrity tests that were performed on the tissue containment system^33^. Full containment system immersion testing was done post-morcellation to ensure that the containment system remained intact and leak-proof after the surgical procedure. For establishing mechanical integrity, puncture and burst tests were performed to evaluate the forces required to cause physical damage to the containment system material during the surgical procedure. However, limited details about the test methodology, acceptance criteria, and test results were provided in the summaries.

Apart from the De Novo and 510(k) summaries, few research studies have provided details about performance testing of tissue containment systems. For example, Anapolski et al.^35^ developed a performance test method to measure the exertion force required to pull TCS from various sized incision ports ranging from 10 to 24 mm in diameter. Their results showed that the mechanical integrity was compromised for incision sizes less than 16 mm. Cohen et al.^36^ evaluated the leakage integrity of various containment systems by morcellating beef tongue stained with dye within a laparoscopic trainer. A visual evaluation was used to locate spilling and leakage of dye through the containment system’s material. Blue dye spill was noted in only one of 12 contained tissue extraction trials. Spillage was visualized from a seam in one of the 4 stitch-sealed rip-stop nylon containment systems before morcellation of the specimen. Similarly, Solima et al.^29^ performed visual examination of the containment system post-morcellation and used spillage of methylene blue as indicator for leakage. Rimbach et al.^37^ evaluated the presence of smooth muscle cells (from beef tongue) from washings in the peritoneal cavity as a measure for the containment system’s leakage integrity. In general, clinical studies used visual examination and dye spillage or peritoneal cavity washings as a measure for evaluating leakage integrity of the containment system. Van Den Haak et al.^38^ performed a Failure Mode and Effective Analysis (FMEA) for contained morcellation and identified ten causes most likely to cause spillage. Of them, two of the causes were TCS material related and could be eliminated by appropriate testing of the TCS materials.

A priori systematic study of the integrity of the containment system is critical and fundamental to ensuring the device performs as intended and provides a mitigation for tissue spread during power morcellation procedures. There is a need for developing (1) a list of potential test methods for safety and performance evaluation, (2) detailed description of the test methodologies, and (3) appropriate acceptance criteria, if any, for those test methods.

The objective of this study is to address these unmet needs by developing a series of preclinical test methods that will assess the mechanical and leakage integrity of the containment system material to mitigate the risk of spillage. The scope of this paper is limited to discussing the mechanical and leakage integrity of the TCS material and not the whole TCS as a device. Henceforth, any reference to TCS in this paper relates only to the TCS material and not the whole device.

To evaluate the mechanical integrity, test-to failure experiments were conducted with the containment systems to evaluate the tensile, burst, and puncture strength of the TCS materials. Similarly, to evaluate the leakage integrity, test-to-failure experiments using dye and bacteriophage as cancer cell surrogates were designed to measure the maximum pressure when the TCS material may leak. Subsequently, materials from different TCS brands were evaluated using these test methods and assessed for correlation between leakage potential and physical characteristics and mechanical strength of the specific TCSs. The novelty of this study is that performance metrics have been compared from each test and correlated with the results of other tests to evaluate TCS material performance as a whole. Based on the test results, several considerations for assessing performance of TCS material during power morcellation are provided.

## Materials and methods

### Materials

A total of seven different brands of TCSs were evaluated experimentally using the test methods described below in Experimental Methods. Summary of the containment systems (with manufacturer information blinded) with material, and thickness details can be found in Table 1. The manufacturers details are blinded for the following reasons. The goal of this study is not to evaluate or qualify the safety or performance of specific brands. Rather, the main goal is to develop generic preclinical test methods that could be used by manufacturers to evaluate the performance of their TCS devices.

**Table 1:** Summary of the different TCSs used in our experiments.

Label	Material	TCS Type	Total thickness (mm)	Thickness of individual layers (mm)	
				*Nylon*	*Polymer*
TC#1	Nylon/PU	Composite	0.141 ± 0.007	0.075 ± 0.015	0.066 ± 0.013
TC#2	Nylon/PU	Composite	0.128 ± 0.002	0.053 ± 0.01	0.075 ± 0.01
TC#3	Nylon/PU	Composite	0.086 ± 0.001	0.055 ± 0.012	0.03 ± 0.012
TC#4	Proprietary	Homogeneous	0.043 ± 0.001	N/A	0.043 ± 0.001
TC#5	PU	Homogeneous	0.108 ± 0.001	N/A	0.108 ± 0.001
TC#6	PU	Homogeneous	0.165 ± 0.005	N/A	0.165 ± 0.005
TC#7	PU	Homogeneous	0.041 ± 0.004	N/A	0.041 ± 0.004

These TCSs are typically made of from either a homogeneous material such as polyurethane (PU) or from a combination of different materials such as rip-stock nylon and PU. Six of the TCSs chosen for testing were described in the literature for clinical use in insufflated contained tissue extractions. The seventh TCS, TC#7, is the only brand in the test samples that was authorized for marketing by FDA as a gynecologic laparoscopic power morcellation containment system.

### Experimental methods

Table 2 lists the test methods that were developed to evaluate mechanical strength and leakage potential of the TCS materials, and to correlate leakage potential with physical characteristics and mechanical strength of the specific TCS materials. The measurement metrics and rationale for these test methods are also provided in Table 2.

**Table 2:** Summary of preclinical test methods for TCSs.

Evaluation type	Preclinical testing	Outcome/metric	Rationale
Physical characteristics evaluation	Material thickness measurement	Material thickness of different layers	To evaluate design requirements for the TCS material through physical characteristics such as thickness and material composition
	Material homogeneity observations	Material structure, morphology, and defects	
Mechanical strength evaluation	Tensile testing	Ultimate tensile strength Toughness	To evaluate strength of the containment system when subjected to tensile, radial, and puncture forces
	Burst testing	Burst pressure	
	Puncture testing	Full puncture force	
Leakage Integrity (or material impermeability) evaluation	Dye penetration testing	Leakage pressure	To understand the ability of the TCS material to remain impermeable under clinically relevant forces
	Microbiological penetration testing	Validation of dye penetration testing	
	Dye penetration testing after partial puncture	Minimum puncture force that caused leakage without puncturing the bag (i.e., partial TCS damage)	

Evaluating the physical characteristics of the TCS material is critical to ensure that they meet the design requirements set forth by the manufacturers. Mechanical strength evaluation is critical to ensure that the material strength of the TCS is not compromised when they are subjected to tensile, radial, and puncture forces arising during the morcellation clinical procedure. Leakage integrity evaluation is critical to ensure that the TCS material, when subjected to clinically relevant forces, can still act as an effective barrier against penetration of tissue, blood and cancer cells. As part of this testing, leakage pressure of the TCS materials were obtained and compared to the pressures experienced during the power morcellation procedure. In addition, the resistance of the device to leakage may be related to its mechanical strength, the material composition, and the thickness.

For each TCS brand, a total of atleast three specimens, each from different samples, were tested to obtain the mean and standard deviation for all quantities of interest for physical characteristics and mechanical strength evaluations as defined in Table 2.

### Physical characteristics evaluation

The containment system material’s thickness was measured using a micrometer (Mitutoyo, Model 293-344-30) which had a minimum resolution of 0.001 mm. The thickness measurements were repeated 5 times at different locations on cut specimens to obtain a mean and standard deviation for each containment system (Table 1). High resolution microscopy was performed with a scanning electron microscope (SEM, Jeol JSM-6390, Jeol USA Inc, Peabody, MA) to evaluate the structure and morphology of all TCSs by observing the inside and outer surfaces, and the cross-section of the TCS material. The fresh TCS specimens were sputter-coated with gold and examined for differences in morphology (e.g., the nylon and the polymer sides of a non-homogeneous tissue containment system) at high vacuum conditions with magnifications ranging from 30× to 800×. In addition to evaluating the structure and morphology of the containment system, the SEM images were also used to obtain thickness measurements of the different layers for composite/non-homogeneous containment systems and to verify thickness measurements made using the calipers for homogeneous containment systems.

### Mechanical strength evaluation

#### Tensile testing

**Figure 1:**
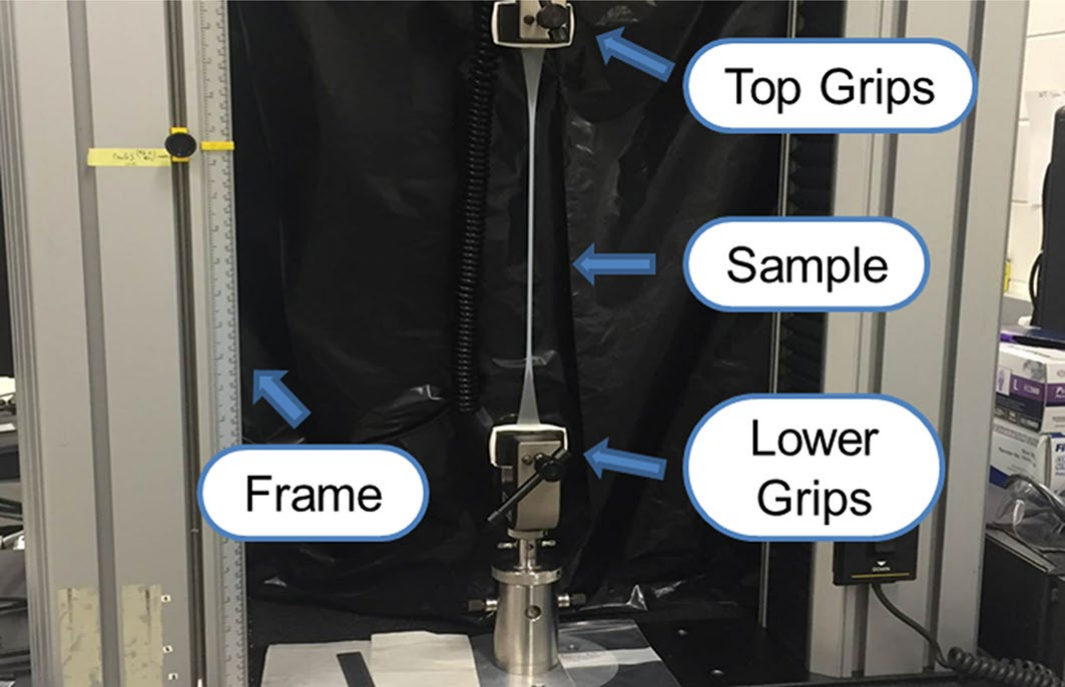
Experimental setup for tensile testing.

The materials testing machine (Instron Tabletop Model 33R4465 & 3300 Controller, Instron Corp., Canton, MA) shown in Fig. 1 was used for performing tensile testing and the force–displacement data collected was used for assessing the stress versus crosshead strain relationship, and estimating the ultimate tensile strength (UTS) and toughness of the TCS materials for all seven brands. TCS specimens were cut with the D412 dog-bone shaped die (Type C—25 mm × 115 mm), mounted on the tensile testing equipment and elongated at 50 mm/min^39^ until the specimen failed. The mean thickness of each specimen was measured at 3 different places and mean thickness along with the sample width was used for calculating stress. From the stress–strain curve, quantities of interest such as UTS (stress at break), and toughness (total area under the stress–strain curve) of each TCS material were estimated.

#### Burst testing

**Figure 2:**
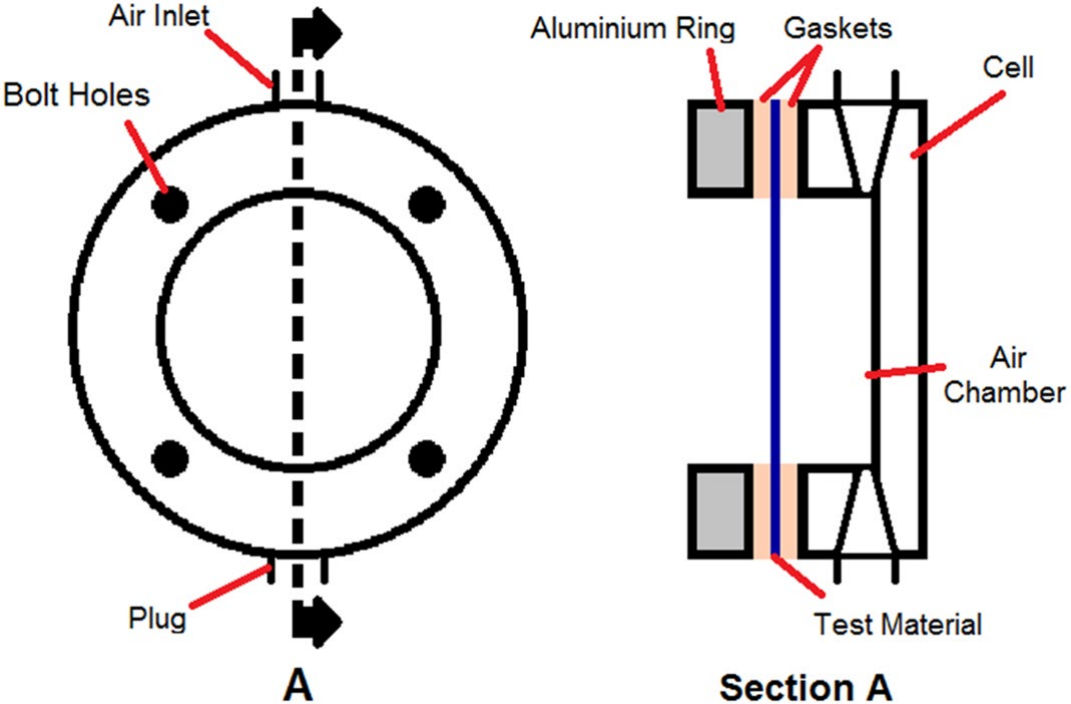
Schematic of the burst testing setup.

A small square coupon (108 mm length) cut out from TCS was used as a test specimen, attached to a circular specimen holder with an internal diameter of 57.3 mm using four screws and connected to an air-pressure chamber^40–42^ (Fig. 2) for burst testing. The test specimen was constrained to allow radially outward expansion (Fig. 2). Compressed air flow into the chamber was controlled with a mass flow controller (Alicat Scientific, MC-20SLPM, Tucson, AZ) and set to allow air into the chamber at a rate of 2 CC/s with the pressure and flow rate being continuously recorded during the test. From the pressure–time relationship, the burst pressure was estimated at the time point where the pressure dropped drastically to atmospheric value.

#### Puncture testing

**Figure 3:**
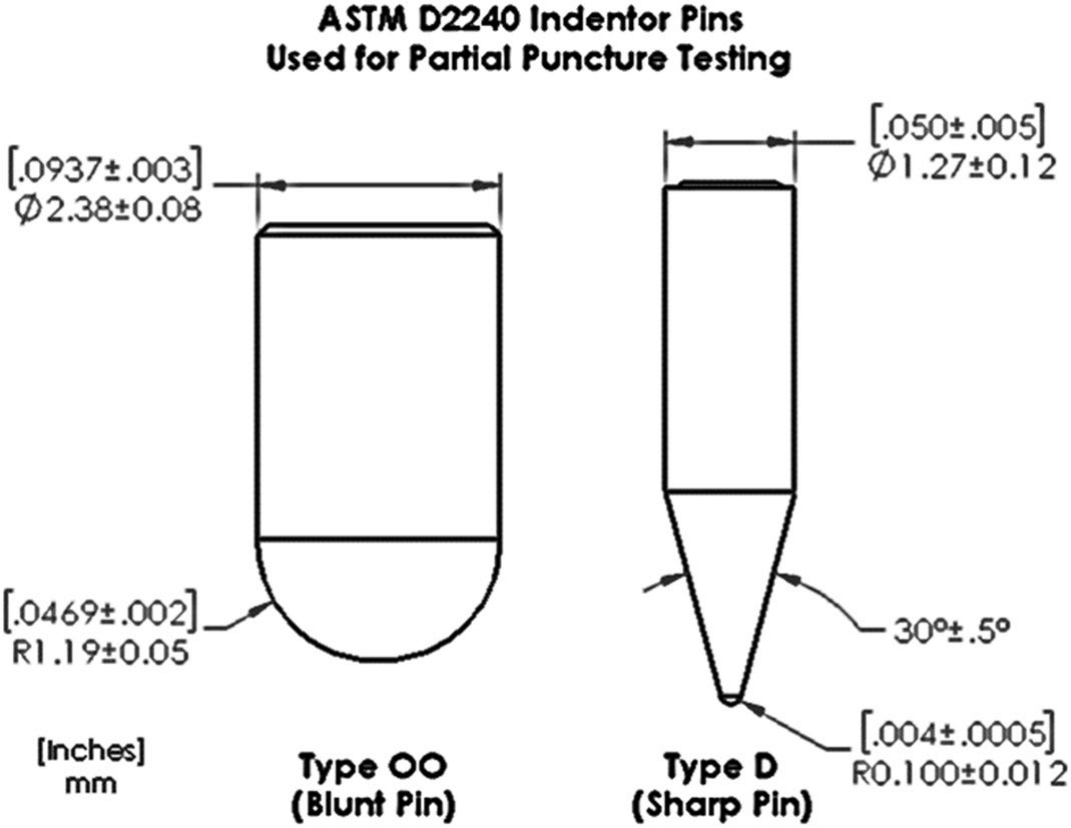
Diagram of the ASTM D2240 durometer pins that were used for the puncture testing in our experiments. Dimensions in brackets are in Inches while the dimentions just below are in millimeters.

**Figure 4:**
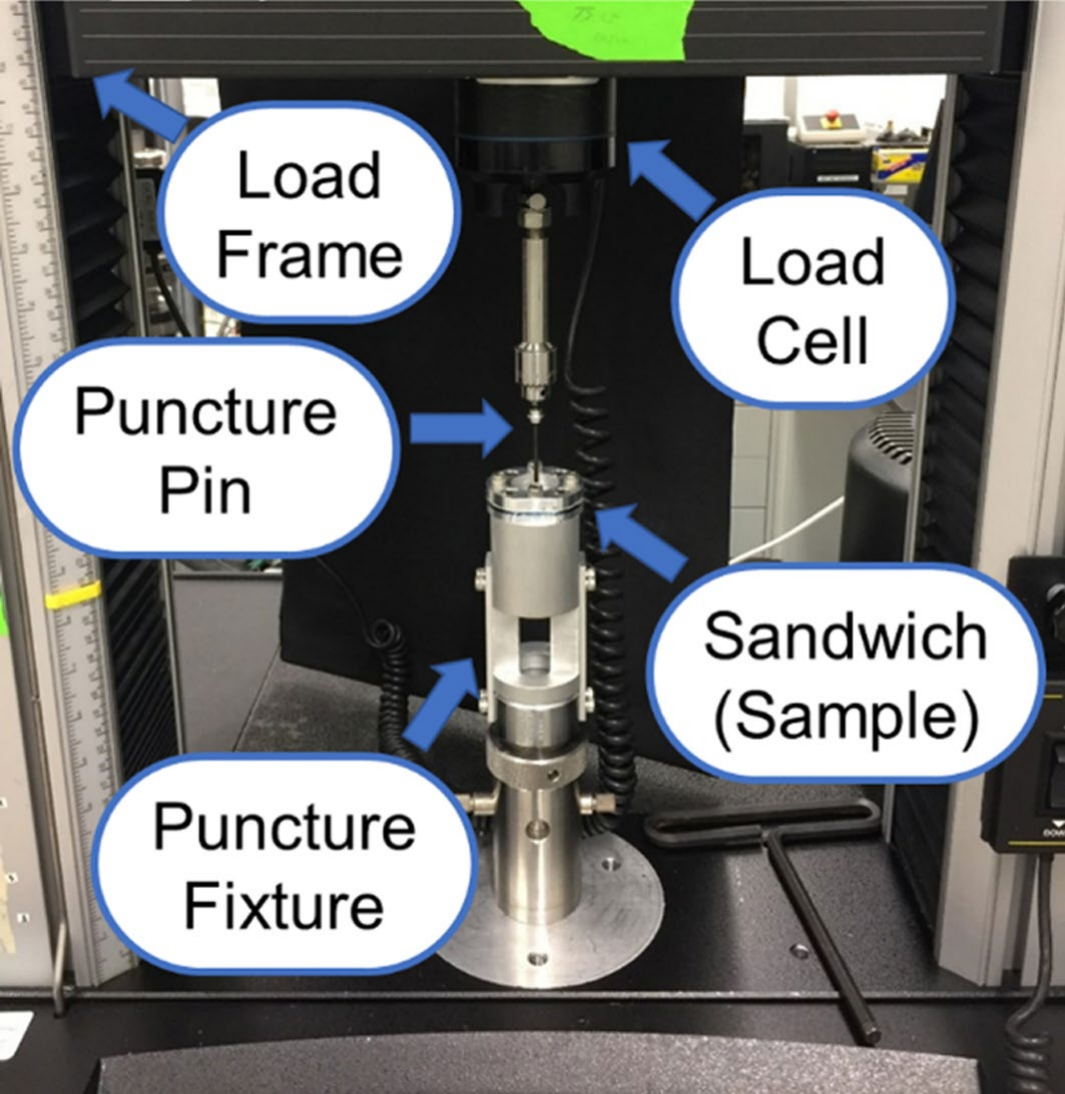
Experimental setup for puncture testing.

The resistance to puncture for each containment system was obtained by measuring the force required to cause a standardized puncture pin to completely penetrate through the thickness of the specimen^43^. A small square coupon (108 mm length) was cut from the TCS and was used as a test specimen sandwiched to a circular specimen holder with an internal test diameter of 25 mm using four screws. The specimen holder was attached to the lower end of the Tabletop Instron (same as above in *Mechanical Strength Evaluation*—*Tensile Testing*). One of the two standard durometers pins, Type OO and Type D^44^ of tip radius 1.19 mm and 0.1 mm (Fig. 3) respectively, were attached to the load cell located on the upper end of the load machine (Fig. 4). The pin moved uniformly downwards with a speed of 25 mm/s until it punctured through the thickness of the containment system while the force–displacement data was recorded. The threshold force at which the pin traversed through the full thickness of the TCS (i.e., fully punctured) was called the full-puncture force (F_puncture_).

### Leakage integrity (or material impermeability) evaluation

#### Dye penetration and microbiological penetration testing

**Figure 5:**
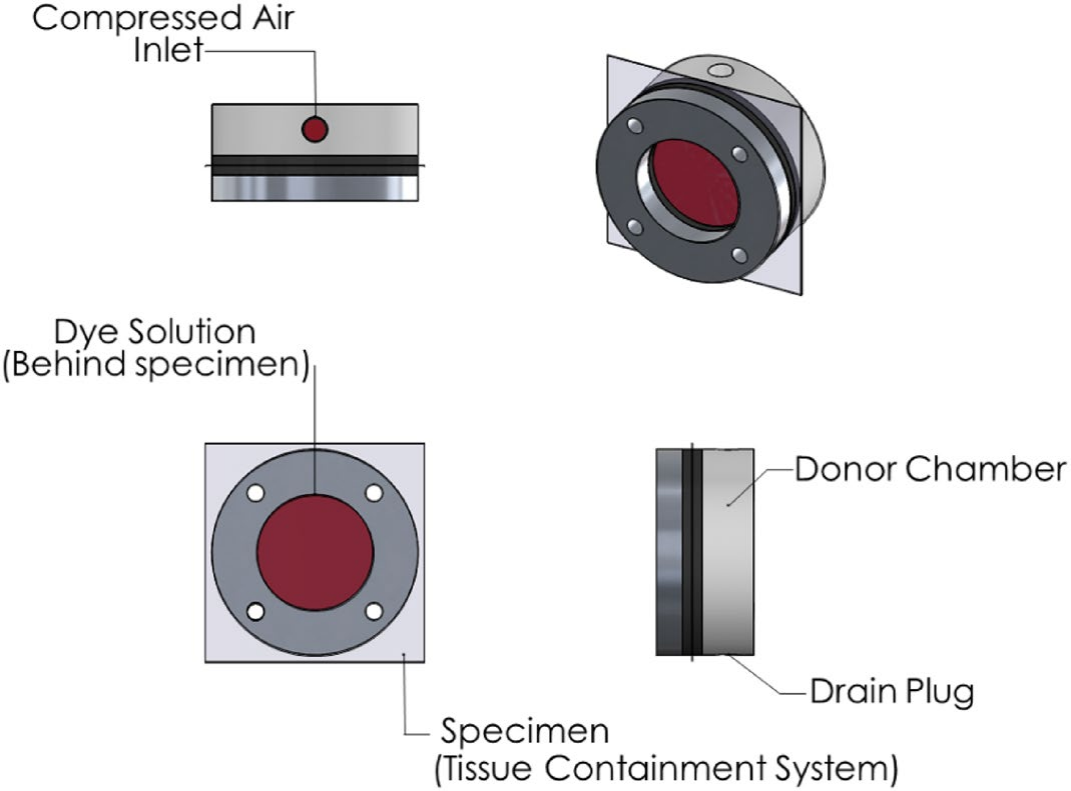
Schematic of the dye penetration set-up.

Dye penetration testing was performed using an ISO blood surrogate dye for determining leakage potential in terms of leakage pressure of different TCS brands (using a minimum of 6 specimens sandwiched in a chamber as shown in Fig. 5). For procedural details on dye penetration testing, please see Herman et al.^45^. The sensitivity of the dye test method was validated using microbiological penetration testing.

Microbiological penetration testing was performed using Bacteriophage Φ-X174 (ATCC 13706-B1) as a surrogate for detecting blood/tissue cell leakage based on its small size, spherical morphology, environmental stability, non-human infectivity, and rapid assay^40^. All experiments were repeated in triplicate. For a test sample to “pass”, all three plates from three specimens should be negative for the presence of plaques which indicates the penetration of the viral surrogate through TCS.

#### Dye penetration testing after partial puncture

**Figure 6:**
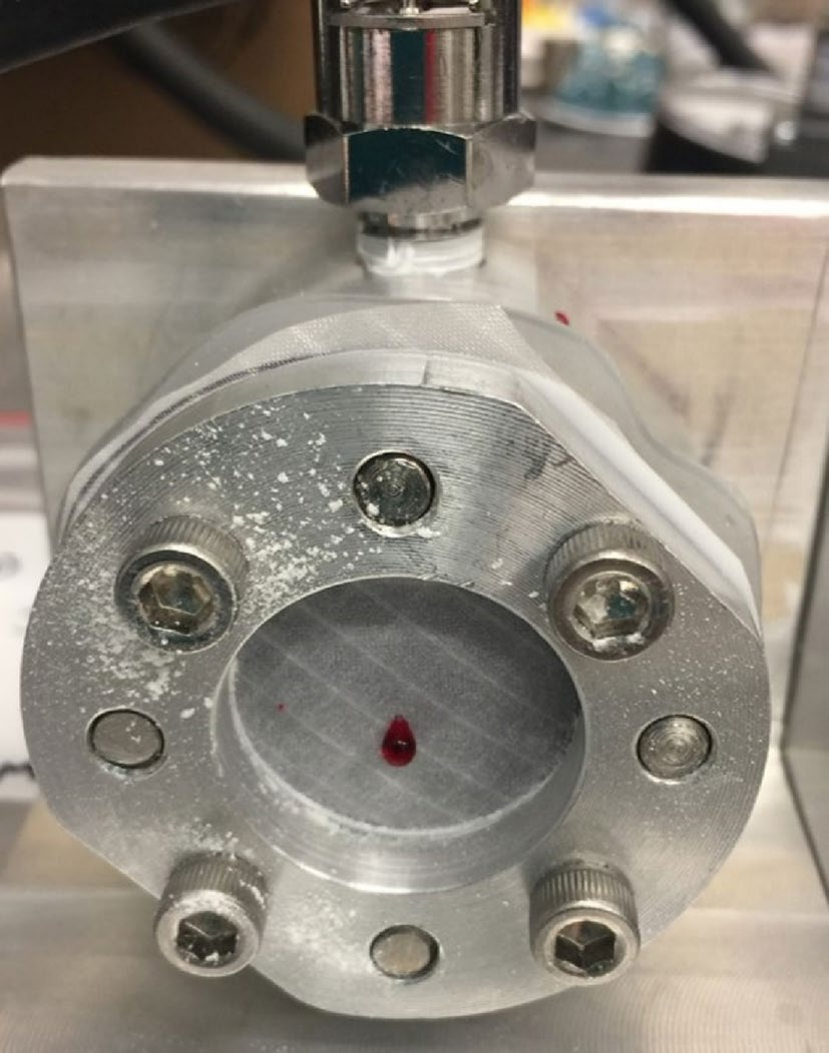
Picture of the dye penetration testing apparatus used after the partial puncture testing.

A combination of partial puncture followed by dye penetration testing was performed to estimate the tool pressure required to cause partial damage to the TCS material but significant enough to cause leakage of the contents. The test setup for puncture and dye penetration testing are the same as shown in Figs. 4 and 5, respectively. The sandwich plate with a circular specimen of diameter of 50 mm (Fig. 6) was designed to be compatible with both the puncture testing rig first and then the dye penetration testing apparatus (Fig. 6).

**Figure 7:**
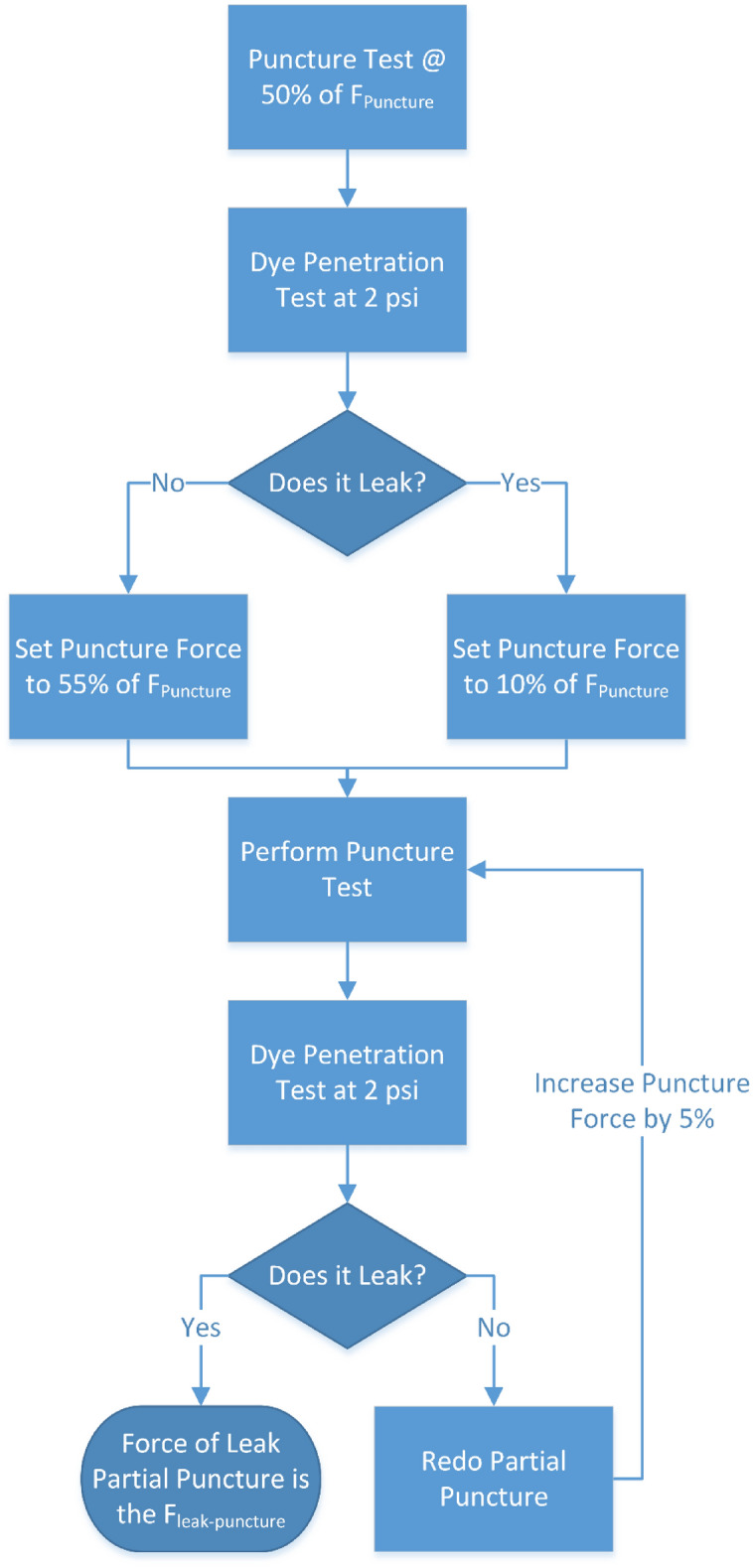
Flow chart describing the partial puncture and subsequent dye penetration testing.

The flow chart outlining the partial puncture study is provided in Fig. 7. After attaching to the puncture testing rig, the TCS specimen was subjected to a force of up to 50% of the full puncture force (0.5 × F_puncture_) using the Type D durometer pin as shown in Fig. 3. Subsequently, the specimen was detached from the puncture testing rig and attached to the dye penetration testing apparatus. The TCS specimen was then exposed to a pressure of 2 psi for 1 min. Although, the insufflation pressure during power morcellation can be up to 0.5 psi, the net instantaneous pressure experienced during the procedure when power morcellation forces are included is of the same order as 2 psi^45^.

If the TCS specimen passed the dye penetration test and deemed to be leak-proof after partial puncture (with 0.5 × F_puncture_), the combination of partial puncture followed by dye penetration testing was repeated for incrementally higher partial puncture forces (at 5% increment) until all the TCS samples of the same brand leaked. However, if the TCS materials leaked at 0.5 × F_puncture_, the combination test was repeated at 0.1 × F_puncture_ and incrementally increased by 5% until TCS samples showed evidence of leak. The force at which the TCS specimen showed first sign of leakage is referred to as the threshold partial puncture force causing leakage (F_leak-puncture_).

## Results

### Physical characteristics evaluation

**Figure 8:**
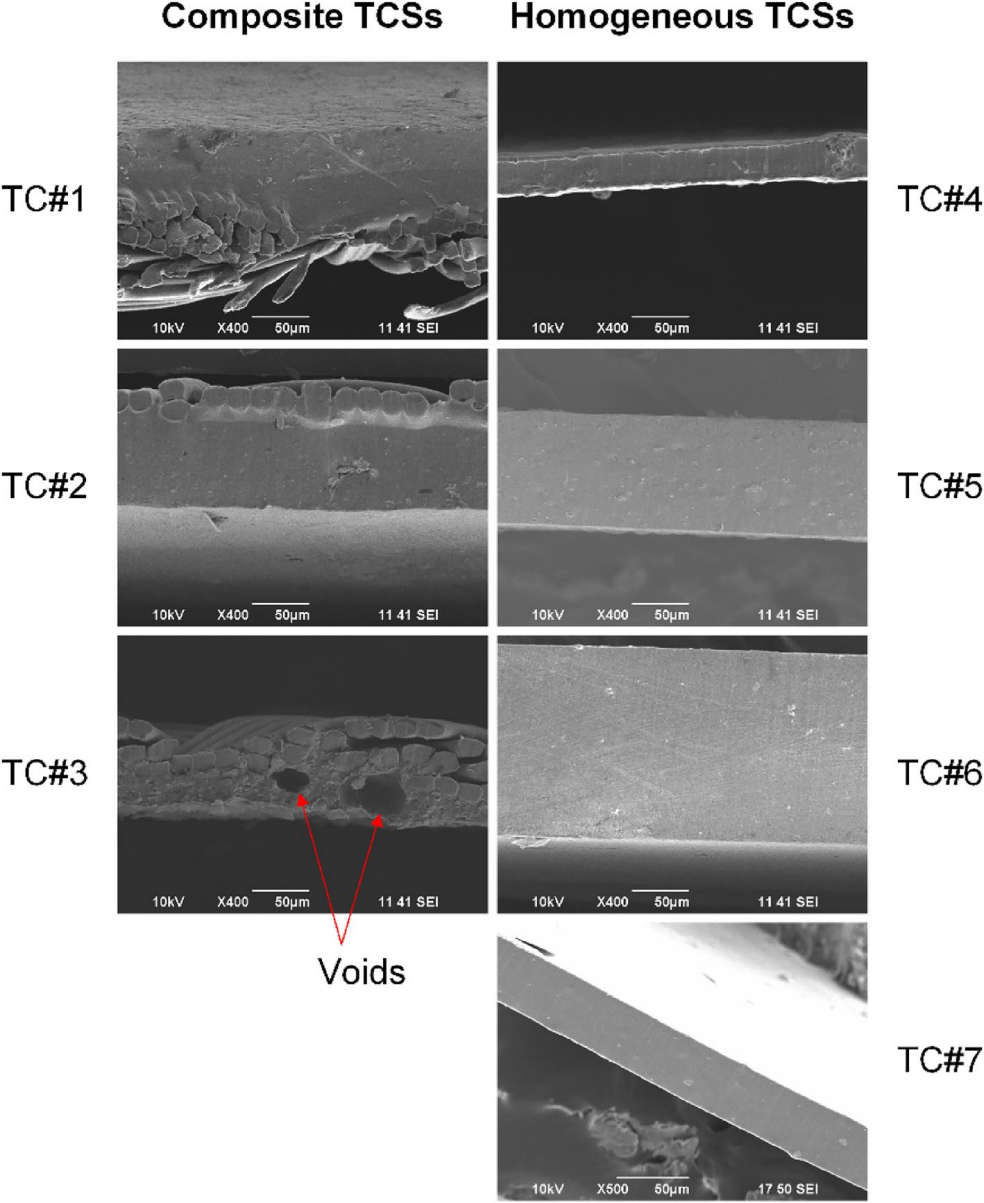
SEM images (cross-sectional view along the thickness) of all seven TCSs.

**Figure 9:**
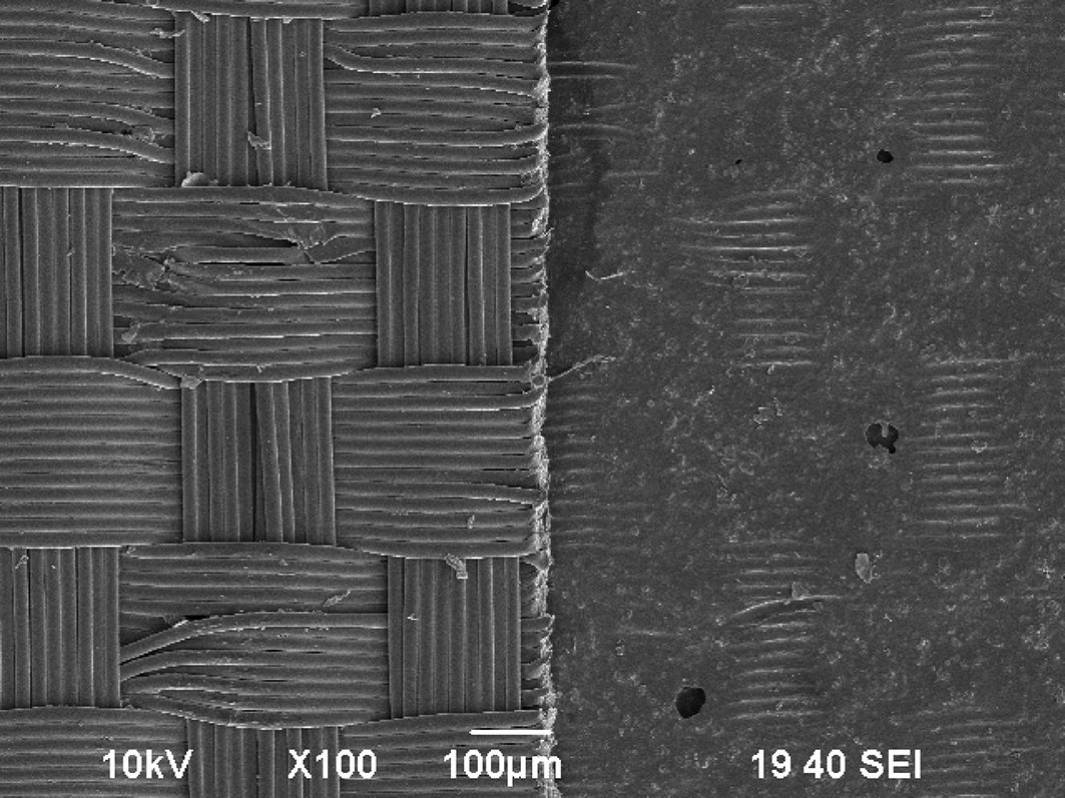
Planar SEM view of TC#3 taken from the nylon side (left) and polymer side (right). On the polymer side the nylon fibers are leaving an impression on the polymer layer in addition to a few voids in the polymer layer.

Thickness of the TCSs varied widely between different brands and ranged from 0.04 to 0.165 mm (Table 1). The composite TCSs presented two distinct layers, Nylon and PU, and contained thinner PU layer compared to other homogeneous TCS. The SEM images showed no differences in the material homogeneity and composition for the homogenous TCSs (Fig. 8). Of all the composite TCSs, TC#3 contained several manufacturing defects/voids in the polymer layer and at the intersection of polymer and nylon fabric. A planer view of TC#3 (Fig. 9) shows that the polymer side was either very thin or completely missing at some locations. Additional details about the SEM data on different TCSs are presented in Herman et al.^45^.

### Mechanical strength evaluation

#### Tensile strength

**Figure 10:**
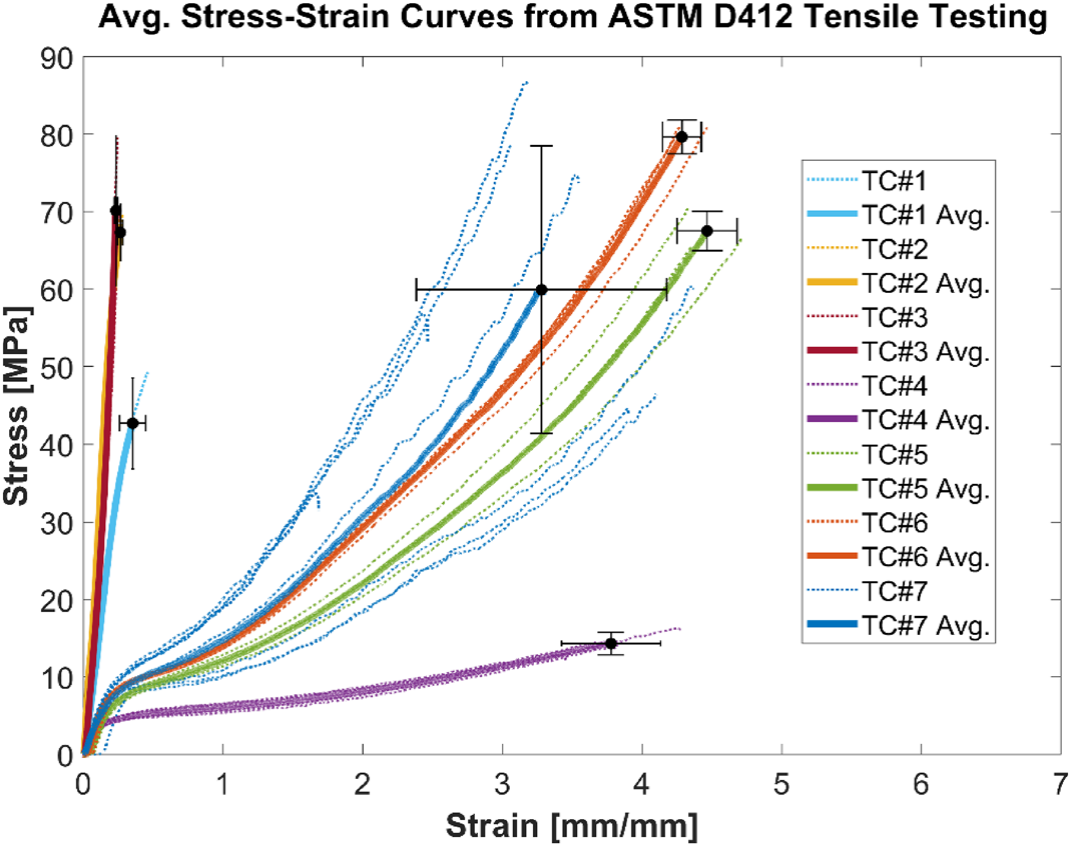
Stress–strain relationship from tensile testing of TCS materials. The thicker brighter lines represent the averaged curve (denoted by ‘Average’ here for each TCS material) from the stress–strain curves of all specimens tested of a specific TCS; the last point on the curve also shows the variation with standard deviation of the stress and strain values obtained from all specimens of a specific TCS material. n = 3 for TC# 1, 2, 3, 5; n = 4 for TC# 4, 6; n = 8 for TC#7.

The stress–strain relationship obtained from tensile testing varied widely between different TCS materials (Fig. 10). All the homogeneous polymer TCS materials (TC#4–7) can withstand larger amount of strain, up to 400%, when compared to the composite TCSs (TC#1–3) which withstood strain values less than 50%. Other metrics for characterizing mechanical strength such as ultimate tensile strength (UTS) and toughness are listed in Table 3. UTS refers to the maximum stress the TCS material can withstand prior to failure while subjected to tensile loading. Toughness measures the ability of a material to absorb energy and plastically deform prior to failure. Depending upon the TCS material, the UTS values varied between 14 and 80 MPa. No correlation was found between the specific type (composite vs homogeneous) of TCS material and the UTS. All composite TCS materials had lower toughness values (< 10 MPa) compared to the homogenous ones (up to 146 MPa). In comparison to TC#7, UTS was higher for TC#2, 3, 5, and 6. Similarly, the toughness was more for TC#5 and 6 than for TC#7. On the contrary, all composite materials had higher Young’s Modulus than the homogeneous materials. Since the focus of this study is to estimate the failure point (UTS) for different TCS materials, yield strength was not estimated.

**Table 3:** Mechanical strength characterization metrics (average and standard deviation) from tensile and burst testing of different TCS materials. P values from one-way ANOVA test comparing TC#7 with rest of the bags are provided in the table.

Label	TCS type	UTS (MPa)		Toughness (MPa)		Max burst pressure (psi)		Youngs modulus (MPa)
		Avg ± Std	P value compared to TC#7	Avg ± Std	P value compared to TC#7	Avg ± Std	P value compared to TC#7	Avg ± Std
TC#1	Composite	42.697 ± 1.446	3.25E−01	8.649 ± 4.339	4.45E−05	65.19 ± 2.02	0.00E + 00	175 ± 0.3
TC#2	Composite	67.303 ± 3.627	9.70E−01	9.736 ± 1.384	5.36E−05	62.50 ± 0.62	0.00E + 00	364 ± 5
TC#3	Composite	70.122 ± 9.654	8.68E−01	6.472 ± 0.947	3.08E−05	77.54 ± 0.67	0.00E + 00	227 ± 15
TC#4	Homogeneous	14.341 ± 5.844	5.05E−05	31.968 ± 4.294	9.37E−04	2.16 ± 0.06	3.29E−02	42 ± 1.2
TC#5	Homogeneous	67.502 ± 2.546	9.66E−01	127.556 ± 5.465	6.36E−02	9.44 ± 0.37	3.94E−05	55 ± 1.7
TC#6	Homogeneous	79.173 ± 2.417	2.52E−01	145.481 ± 5.169	2.77E−03	14.67 ± 0.51	4.32E−09	78 ± 6
TC#7	Homogeneous	60.283 ± 18.552	N/A	88.73 ± 30.302	N/A	4.45 ± 0.16	N/A	64 ± 4.2

#### Burst testing

**Figure 11:**
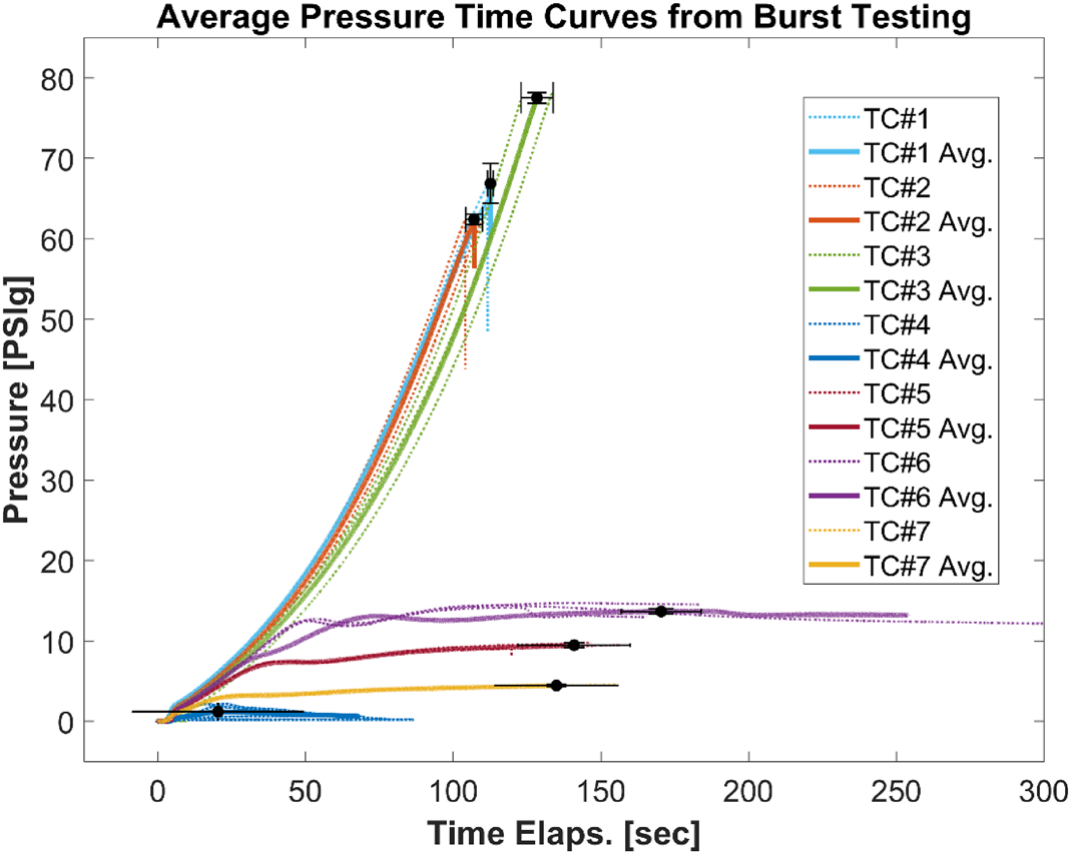
Air pressure as a function of time for different containment systems during burst testing. The thicker brighter lines represent the averaged curve from the pressure–time curves of all specimens tested of a certain containment system; the last point on the curve also shows the variation with standard deviation of the pressure and time values obtained from all specimens of a certain containment system. n = 3 for all TCSs except TC# 4 (n = 4).

The pressure rise response to radial loading was distinctly different for homogeneous and composite TCS materials (Fig. 11). For the composite TCS materials, the pressure inside the TCS steadily increased with time until it reached the point of full burst. However, for homogeneous TCS materials, the pressure steadily increased with time up to the yield point (of elastic expansion) for the TCS material. Subsequently, the TCS volume increased at a faster rate (i.e., plastic deformation) causing the TCS pressure to either stabilize or drop slightly depending upon the TCS material and thickness.

Similar to the UTS and toughness values, the burst pressure (P_burst_) values varied widely between the TCS materials and ranged from 2 to 78 psi (see Table 3). Burst pressure refers to the maximum radial stress the TCS material can withstand prior to failure. All the homogeneous TCS materials had lower P_burst_ values (< 15 psi) compared to composite ones (up to 78 psi). In comparison to TC#7, burst pressure was higher for TC#1, 2, 3, 5, and 6.

#### Puncture testing

**Figure 12:**
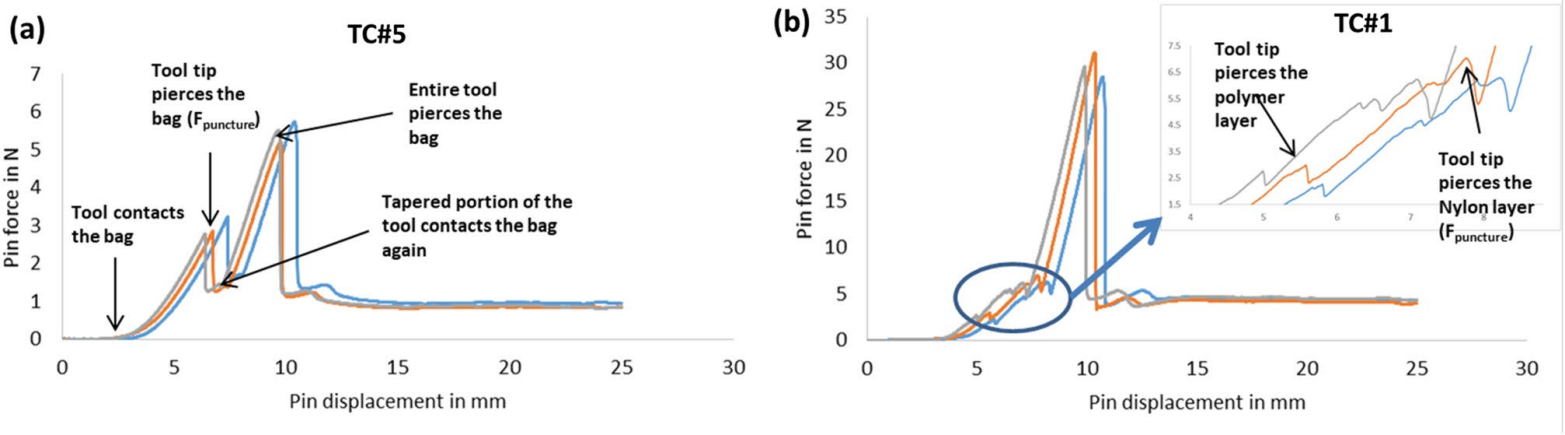
The force exerted by the type D durometer pins as a function of compressive displacement of the TCS specimen for two different TCSs.

Figure 12 shows the force exerted by the durometer pin (D) on homogeneous and composite TCS materials as the pin is pushed through them. For the homogeneous TCS materials (Fig. 12a), once the pin contacts the TCS, the force increases with time until the pin’s tip punctures the TCS. This force at which the pin’s tip pierces the TCS is determined to be the puncture force (F_puncture_). After puncturing the TCS, the force drops suddenly as the pin’s tip is no longer in contact with the TCS. As the pin displaces further at a uniform speed, the tapered part of the pin’s tip contacts the TCS causing the tool force to increase with the displacement of the pin. The force keeps increasing until the distal part of the pin geometry penetrates completely through the TCS material, however, the forces post F_puncture_ were not considered for further analysis. Instead of a single spike in the force curve seen for the homogeneous TCSs (Fig. 12a), the composite TCS materials show two small spikes representing penetration through the individual layers in the composite containment system (inset in Fig. 12b). The first spike occurs when the pin’s tip punctures through the thin polymer layer like the homogenous TCSs. The second spike occurs when the pin’s tip punctures through the Nylon fabric. The force at which the pin’s tip pierces both layers of the TCS specimen is determined to be the puncture force (F_puncture_) for composite TCS materials.

**Figure 13:**
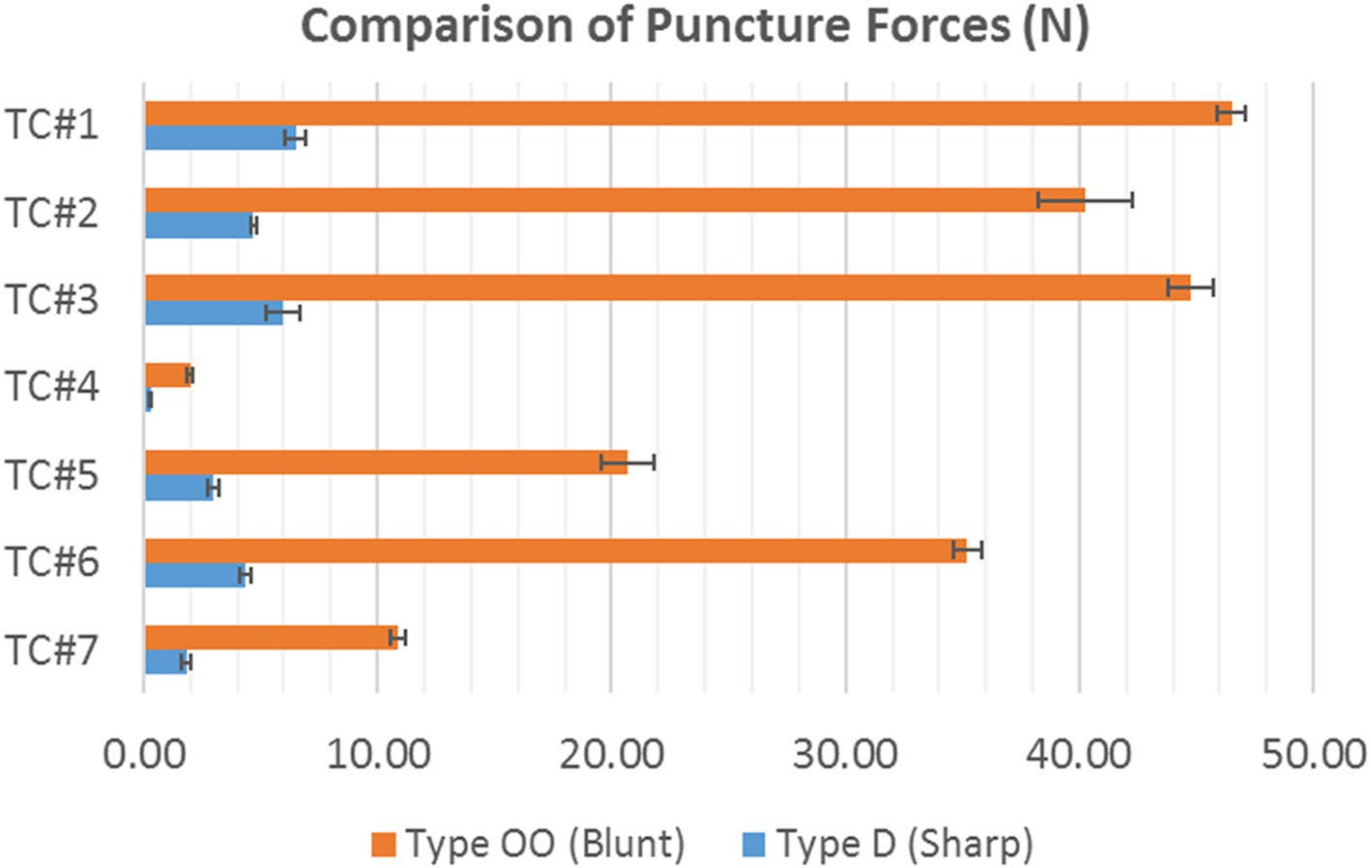
Maximum force to puncture (F_puncture_) for different containment systems from puncture testing using Type OO blunt and Type D sharp durometer pins with one standard deviation error bars. n = 3 for all TCSs.

Figure 13 shows the puncture force for all TCS brands obtained for Type OO and D durometer pins. F_puncture_ was an order of magnitude more for Type OO blunt pin compared to Type D sharp pin. F_puncture_ was higher for composite TCS materials (40 N–45 N) compared to homogeneous TCSs (2.5 N–35 N) for Type OO blunt pin. Same observation was made for Type D sharp pin (4–7 N vs. 0.5–4 N for composite vs. homogeneous TCS materials respectively). For the homogeneous TCSs made of the same material (PU), the F_puncture_ increased proportionately with the TCS thickness. In comparison to TC#7, the force at puncture for both types of pins were higher for all other TCSs except TC#4.

### Leakage integrity (or material impermeability) evaluation

#### Dye penetration and microbiological penetration testing

The dye and microbiological penetration in different TCS materials were reported previously in Herman et al.^45^. In summary, when exposed to an external pressure of 2 psi, all the TCS materials except TC#3 remained leak proof during both dye and microbiological penetration testing. For TC#3, ~ 89% and ~ 58% of the tested TCS specimens failed (i.e., leaked) the dye and microbiological penetration testing, respectively.

Unlike the mechanical testing results, no clear trend was observed for the leakage potential (with leakage pressures P_leakage_) performed previously when comparing the homogeneous and composite TCSs (Table 4). Of all the tested TCS brands, only TC#3, which is a composite TCS, leaked at 2 psi. Other composite TCSs like TC#1 and 2 did not leak at 2 psi. On the contrary, none of the homogeneous TCSs exhibited leakage at 2 psi. In comparison to TC#7, P_leakage_ was higher for all other TCS specimens except TC#3 and TC#4.

**Table 4:** Leakage potential (negative or positive) at 2 psi obtained from both dye and microbiological penetration testing performed previously. Leakage pressures were obtained from previous dye penetration tests^40^.

Label	Material	Leakage at 2 psi^40^	Leakage pressure (psi)^45^
TC#1	Nylon/PU	Negative	> 25
TC#2	Nylon/PU	Negative	> 25
TC#3	Nylon/PU	Positive	0.5
TC#4	Proprietary	Negative	2.2
TC#5	PU	Negative	> 6
TC#6	PU	Negative	> 10
TC#7	PU	Negative	4

#### Dye penetration testing after partial puncture

**Figure 14:**
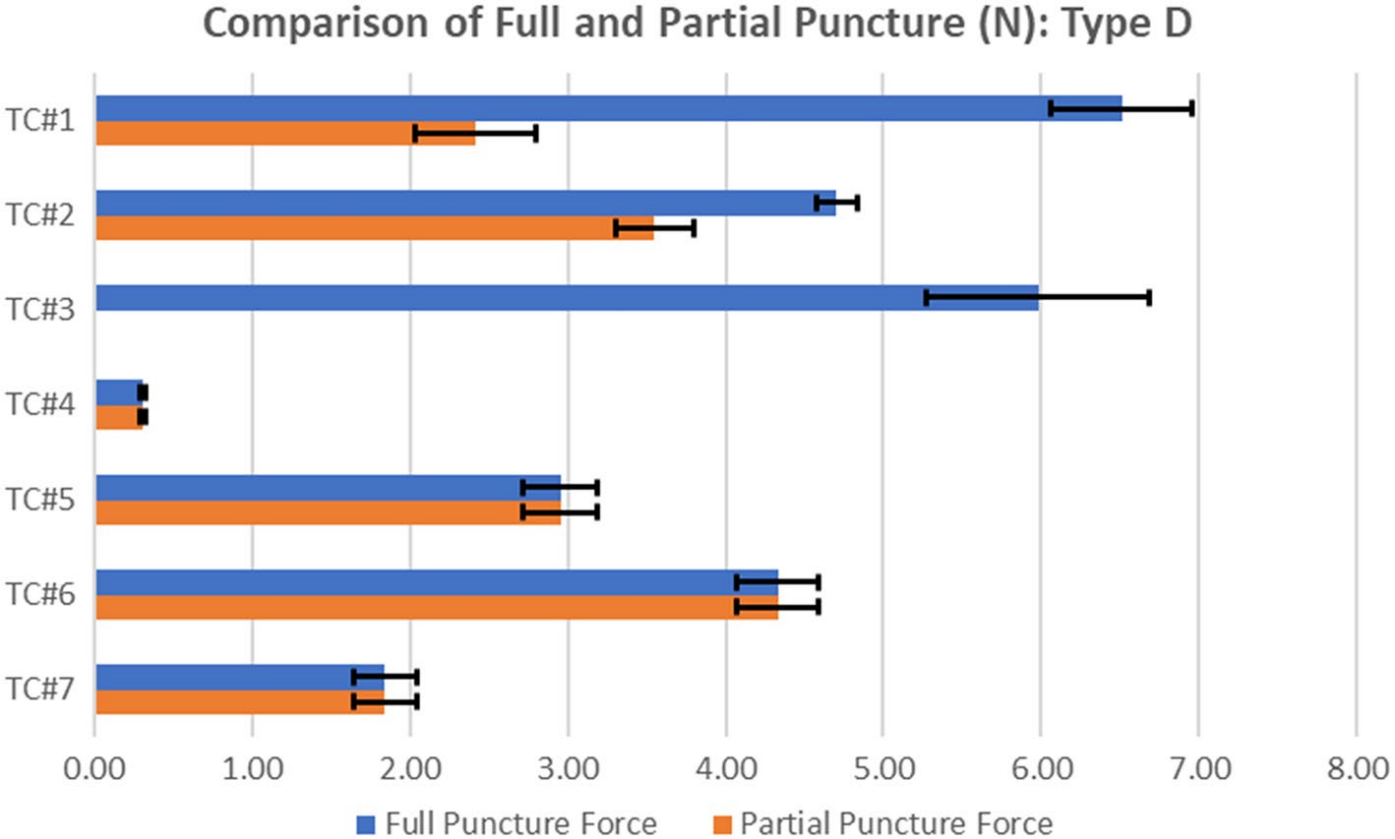
Partial puncture leak force, F_leak-puncture,_ compared to the full puncture force, F_puncture_, for Type D Pin; n = 3 for all TCSs during full puncture testing; For partial puncture testing, n = 3 for TC#4–7, and n = 10 for TC#1&2.

For the composite TCS materials, the F_leak-puncture_, which is the force at which the TCS specimen was partially damaged (and not fully punctured) and caused leakage of its content, was significantly lower in comparison to F_puncture_ values (Fig. 14). TC#1 and TC#2 first started leaking once the pin force was 35% and 65% of F_puncture_, respectively with an average force of 37% and 75% of F_puncture_ for TC#1 and TC#2 respectively. TC#3 leaked without applying any puncture force and F_leak-puncture_ was assigned to be 0%. For homogeneous TCS specimens, since the TCS material contained only the polymer layer, the F_puncture_ and F_leak-puncture_ was assumed to be the same (i.e., the TCS material leaks only when the polymer layer is fully punctured. To confirm this, partial puncture testing followed by dye penetration testing was performed for up to 85% of F_puncture_ for TC#7, and no leaks were observed. In comparison to TC#7, F_leak-puncture_ was higher for all other TCS materials except TC#4.

## Discussion

Preclinical testing requirements for TCSs include, but are not limited to, evaluating TCS’s material impermeability to tissues, cells, and fluids, and demonstrating device integrity when used with intended laparoscopic instruments and morcellators^13,46^. Our study developed test methods to evaluate different TCS materials and provided a structured framework to correlate leakage potential with physical characteristics and mechanical strength of the specific TCS materials. These methodologies may be useful for stakeholders, such as medical device manufacturers, test laboratories, academic researchers, and regulatory bodies while evaluating the performance of various TCS materials. Since only a small specimen of TCS material was tested instead of the whole TCS bag, additional test methods need to be developed for evaluating the leakage integrity of whole TCSs (i.e., as a full finished medical device).

As a part of this study, TCS materials were exposed to tensile, radial, and puncture forces to represent physiologically relevant loads anticipated during clinical use. For example^38^, the TCS experience tensile stress when (1) it is pushed through the trocar into the pneumoperitoneum (i.e. distended abdomen), (2) when maneuvering the TCS in the pneumoperitoneum using graspers, (3) when pulling the TCS out of the abdominal cavity with blood or tissue remains inside it, etc. Similarly, the TCS is subjected to radial stress (1) during insufflation, and (2) during removal of TCS out of the cavity through the narrow incision if the insufflated CO_2_ is trapped inside it. The TCS is also subjected to puncture forces every time the grasper, tenaculum, or the morcellator contacts the device surface.

Our results showed a clear distinction in mechanical performance between composite and homogeneous TCS materials. Overall, materials from composite TCSs showed superior performance for mechanical strength compared to homogenous TCSs, due to the fact that composite TCS materials had higher puncture and burst resistance than homogeneous TCSs (Table 3). On the contrary, homogeneous TCS materials showed increased toughness due to the ability to withstand larger strain than the composite TCSs. Similarly, when the mechanical load is applied, the temporal response of composite and homogenous TCS materials were different as depicted in the stress–strain, burst pressure, and puncture responses (Figs. 10, 11, and 12).

In comparison to TC#7, ultimate tensile strength was higher for TC#2, 3, 5, and 6. Similarly, the toughness was higher for TC#5 and 6 than for TC#7. Burst pressure was higher for TC#1, 2, 3, 5, and 6 than TC#7. The force at puncture for both types of pins was higher for TC#1, 2, 3, 5, and 6 than TC#7. Overall, the mechanical performance was very different for homogenous TCS material compared to composite TCS. Device manufacturers and testing laboratories should be cognizant of these distinctions while comparing the performance of TCS materials. Because of the underlying differences in mechanical performance, a homogenous TCS material may not be the best comparator if the device under evaluation is made of composite layers and vice versa.

Our results also showed that the generally enhanced mechanical strength of the composite TCS materials did not necessarily translate to better leakage integrity with dye penetration testing. This is evident by comparing the leakage pressure of different TCS specimens with their respective mechanical performance. TC#3, which had the maximum burst pressure, and second best UTS and F_puncture_, had the worst leakage pressure and F_leak-puncture_ (Tables 3 and 4 and Fig. 14); therefore TC#3 was deemed the worst in terms of leakage integrity (or material impermeability). The leakage pressure was 50 times less for TC#3 compared to some of the other composite and homogeneous TCS materials (Table 4). As a matter of fact, the leakage pressure of the other composite bag materials TC#1 and TC#2 were much higher than for TC#3. For composite TCSs, the polymer layer keeps the TCS impermeable to cells while the nylon fabric provides the mechanical strength to the TCS. The leakage integrity of the composite materials TC#1 and TC#2 likely is contributed from having at least two times thicker polymer layers than TC#1 (Table 1). While using a composite material, there is a possibility of damage to the polymer layer during the surgical procedure. The damage to the polymer layer may not affect the overall strength and shape integrity of these TCSs but can compromise the leakage integrity. Similarly, different manufacturing processes among different brands can lead to varying physical characteristics of the polymer layers; some processes can be more prone to manufacturing defects such as voids in composite TCSs (TC#3, Figs. 8 and 9) through which contents may leak and compromise the leakage integrity of the TCS. Stakeholders should be aware of this while designing and testing TCS material, and clinicians should be aware of this when selecting composite TCS material for use. Several of the TCS evaluated in in vivo clinical studies are made of nylon-polymer composite materials^18,21–23,25,30^. It is critical to measure the thickness of the polymer layer, leakage pressures, and determine appropriate acceptance criteria for them during device design and verification testing of these device materials. Among the three polyurethane based homogenous TCS, some correlation was observed between material thickness and mechanical and leakage strengths. This suggests that if the TCS of same type and material is chosen as a comparator device, the acceptance criteria for mechanical strength could be developed based on the comparator device material. However, acceptance criteria for a test should also provide sufficient safety factor from worse case clinical use conditions, e.g., in this study leakage performance was evaluated at a minimum pressure of 2 psi which is four times higher than the nominal insufflation pressure of 0.5 psi used clinically. TC#3 failed the leakage integrity testing at 2 psi (Table 4), however the maximum leakage pressure for TC#3 was 0.5 psi showing that this TCS material did not provide any factor of safety against inadvertent surgical procedural issues. These results highlight that stakeholders should consider not only mechanical strength but also the leakage integrity (or material impermeability) when forces from clinical use are applied and establish adequate acceptance criteria and safety factors for the preclinical bench tests.

Leakage integrity was evaluated using dye and microbiological penetration testing. From previous studies^45^, when exposed to an external pressure of 2 psi, TC#3 was the only containment system where ~ 60% and ~ 89% of the tested TCS specimens failed (i.e., leaked) the dye and microbiological penetration testing, respectively. On one hand, the same TCS brand failed in both leakage integrity tests indicating the results of microbiological penetration test and dye penetration test may be similar. On the contrary, the sensitivity for leakage of the microbiological penetration test appears to be higher than the dye penetration test. The results may indicate that microbiological penetration test may be preferred over dye penetration test. Dye penetration testing alone was used in this study for evaluating leakage in partially punctured TCS material and results were compared to previously performed leakage integrity tests. Device manufacturers might benefit from primarily using dye penetration testing to verify leakage performance for different design variations of the TCS material to finalize the design, after which microbiological penetration testing may be performed for validation.

Inadvertent damage to TCS due to contact with surgical tools and morcellators during the surgical procedure is listed as one of the primary risks to patient health with the use of gynecologic laparoscopic power morcellation^33^. While evaluating the puncture resistance of various TCS materials, our studies showed a significant difference between the puncture force required to cause complete penetration through the device thickness (F_puncture_) being as high as two and half times the partial puncture force required to cause leakage (F_leak-puncture_) for the composite materials. The puncture force required to cause complete penetration through the thickness and partial puncture to cause leakage were the same for all homogeneous TCSs indicating breach through the entire thickness of the TCS material in both cases. Consequently, these results showed that partial damage to composite TCS material due to contact with the surgical tools if not noticed could lead to catastrophic leakage of contents out of the TCS. This risk should be taken into consideration while establishing the acceptance criteria for tool forces exerted on the TCS. For this study, all the puncture testing was done using standardized durometer pins (Type OO and Type D in Fig. 3) as penetration tools to enable better comparisons between devices. During device preclinical evaluation, in lieu of these pins, the manufacturers should consider use of real surgical tools or pins that mimic the sharpness, hardness, and contact surface of the surgical tools used during the surgery, as differences in geometry may affect results. Our test methodology demonstrated results by testing a plain specimen cut from all seven TCS brands. During actual device development testing, TCS specimens from multiple manufacturing lots and locations highly susceptible to mechanical failure and leakage such as seams, and junction points (if any) should also be considered, as there may be variability introduced in manufacturing that should be captured.

As a part of this study, several metrics for material performance such as UTS, P_burst_, P_leakage_, F_puncture_ and F_leak-puncture_ were established and measured. However, we did not come up with a set acceptance criterion for any of these metrics. The acceptance criteria tend to be device and material specific and could depend on several factors such as device design, indications for use, choice of comparator device, etc. However, it would be beneficial for the manufacturers to derive appropriate device safety factors^45^ by comparing the device’s test-to-failure metrics obtained after bench testing with clinically relevant forces encountered by the TCS material during actual surgical procedures. Previous studies have used force sensors to measure the force imparted by the clinicians on the surgical tools during medical procedures. The safety factors, if mentioned in product labeling, could help researchers and regulators understand the risk associated with the use of different TCS materials during power morcellation procedures.

The study has following limitations. This study outlined test methods to understand the performance of materials used to make TCSs. However, these protocols, as is, are not applicable to testing of the TCS bag as a whole. Additional test methods are needed to evaluate the mechanical and leakage integrity of the full TCS bag. Further more, additional test methods are needed to evaluate the ability of the device to allow for insertion and removal instruments while maintaining pneumoperitoneum, ability of the intended users to adequately deploy and use the device without compromising its integrity etc. These tests may necessitate the conduct of additional bench testing, clinical simulation, whole device TCS leakage testing, and animal studies to demonstrate performance.

Currently, there is a clear need for developing standardized protocol to evaluate the mechanical performance and leakage potential of the TCS materials. Our study addressed this need my developing a series of bench tests that will be useful in evaluating performance of TCS materials for power morcellation procedures. Streamlining of preclinical testing will create efficiencies in bringing new tissue containment technologies to market, resulting in benefit to both patients and health care professionals.

## Data Availability

The datasets used and/or analyzed during the current study available from the corresponding author on reasonable request.
